# Shifts in sex-specific immune gene expression in a beetle with parental care

**DOI:** 10.1038/s41598-025-95268-4

**Published:** 2025-03-29

**Authors:** Nikoletta A. Nagy, José O. Valdebenito, Johanna Lévai-Kiss, Zoltán Rádai, András Kosztolányi, Tamás Székely, Zoltán Barta

**Affiliations:** 1 Department of Evolutionary Zoology and Human Biology, University of Debrecen, Egyetem Tér 1, 4032 Debrecen, Hungary; 2 HUN-REN-UD Behavioural Ecology Research Group, Department of Evolutionary Zoology, University of Debrecen, Debrecen, Hungary; 3 Facultad de Medicina Veterinaria y Agronomía, Universidad de Las Américas, Concepción, Chile; 4 Instituto Milenio Biodiversidad de Ecosistemas Antárticos y Subantárticos (BASE), Santiago, Chile; 5 One Health Institute, Faculty of Health Sciences, University of Debrecen, Debrecen, Hungary; 6 Department of Dermatology, Medical Faculty, University Hospital Duesseldorf, Heinrich-Heine University, Duesseldorf, Germany; 7 Department of Zoology, University of Veterinary Medicine Budapest, Budapest, Hungary; 8 Milner Centre for Evolution, Department of Biology and Biochemistry, University of Bath, Bath, UK; 9 Present Address: Departamento de Zoología, Universidad de Concepción, Concepción, Chile

**Keywords:** Geotrupidae, *Lethrus apterus*, Transcriptomics, Sexual selection, Sex-bias, Immunoecology, Immune trade-off, Gene expression, Gene expression analysis, Ecology, Immunology

## Abstract

Males and females generally differ in resource investment strategies in order to maximise reproductive output. These strategies involve the control of important systemic processes such as self-maintenance and immune activity, which in turn could be traded-off against aspects of reproduction in a sex-specific manner. While some aspects of this immunomodulation have been previously shown in domestic animals, sex-specific immune modulation using repeated sampling over the breeding period has rarely been tested in the wild. Here we used *Lethrus apterus*, a sexually dimorphic beetle with parental care, to investigate the association between sex roles (e.g. offspring provisioning) and sex-specific immune gene expression. By determining the immune gene activation of males and females at five successive moments within the active season, we found that their sex-specific immune gene expression varies substantially across the active season, alternating between male bias to female bias and vice versa. Though, when pooling all sampling dates together, there was no overall difference in the number of up-regulated immune genes between the sexes. Sex roles in this beetle are associated with energetically demanding behaviours that could potentially explain our results. We highlight the importance of successive sampling protocols to understand ecological dynamics in the wild.

## Introduction

To counteract pathogens and fend off infections, hosts rely on a competent immune system^1^. The immune system is crucial for survival, and evidence shows that its maintenance and function are energetically demanding^2^. In nature, where resources are limited, aspects of immune function may be traded off against other competing physiological and behavioural processes. These trade-offs can result in both up- and down-regulation of immune activity. Such adjustments are particularly noticeable during challenging stages of an animal’s life, such as reproduction, migration or under harsh environmental conditions^3–6^.

In sexually reproducing animals the breeding period is regarded as strenuous and energetically costly, as it may encompass physiological and behavioural processes such as egg production, offspring feeding, and offspring defence^7,8^. Many of these activities are also sex-specific (termed sex roles), and imply shifts in resource allocation within the breeding period that could differentially influence immune activity between the sexes. Accordingly, there is substantial evidence supporting immune modulation associated with parental provisioning in mammals and birds (reviewed in Alonso-Alvarez and Velando^9^). In wild invertebrates, although evidence is considerably less common, the few available studies do recognise shifts in immune function in response to events taking place during reproduction^10,11^. For example, Wang and Liu^11^ found that males of the clam *Meretrix petechialis* exhibited a prominent down-regulation of immune genes after spawning, whereas females showed an up-regulation of a completely different group of immune genes. In *Lethrus apterus*, a beetle which exhibits biparental care and a brief reproductive season (hereafter, active period), Kiss et al.^12^ highlighted a difference in antimicrobial capacity but not in encapsulation ability between the sexes, potentially linked to their investment in reproduction and parental care. The latter study also observed seasonal variation of antimicrobial capacity in females, but it is important to note that only measures taken early in their active period versus near the end were considered^12^. Given the complexity of the breeding season in insects, including *L. apterus*, and how rapidly immune function can be up- or down-regulated, it is possible that sex-specific shifts in immune function may occur numerous times within the reproductive season (as already described, e.g. in birds^13^). However, it is yet unknown how frequently these shifts may occur in invertebrates and how they may relate to the specific sex roles displayed within the breeding period. Importantly, though determining specific immune parameters such as antimicrobial capacity or encapsulation can be informative, such methods are limited in the depth of information they provide, overlooking other important components of insect immunity, such as antimicrobial peptides (AMPs) or lysozymes^14^. This limitation can be addressed by using other specialised and more comprehensive techniques, such as transcriptome analysis, which can provide a much more in-depth understanding of the insect′s immune response at the molecular level^15^.

**Fig. 1:**
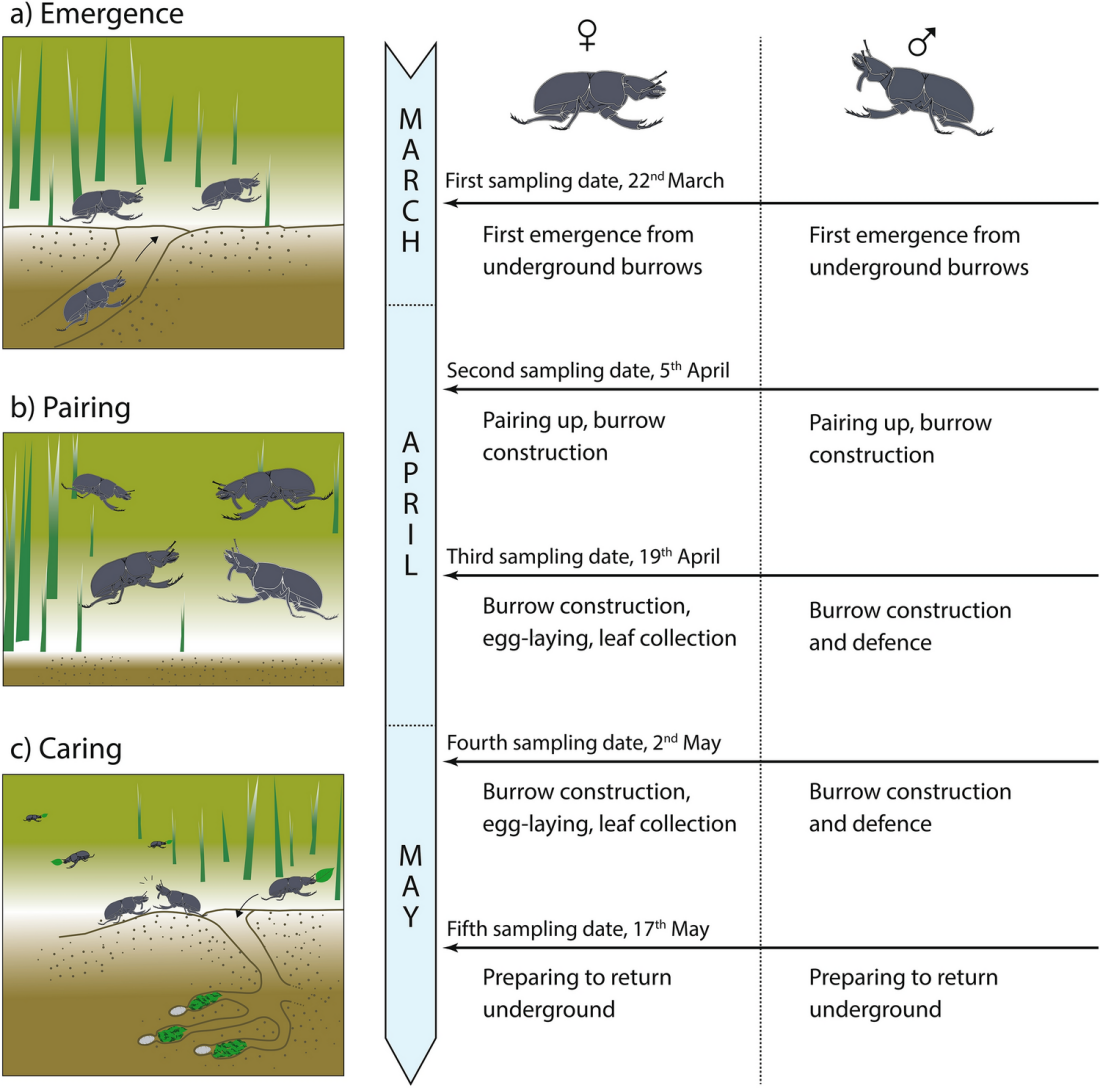
Schematic summary of the active period in* Lethrus apterus*, also showing the corresponding sampling dates and predominant sex roles. (**a**) In early March, the beetles start emerging from their underground burrows to (**b**) find a mate and reproduce. In April, the caring stage starts, where the sexes display rather distinct sex roles (**c**). Mostly females collect leaves to provide the offspring with sufficient food for development, while males generally guard the entrance of the nest from both conspecific and heterospecific intruders. The main sexually dimorphic trait is the mandibular processes (tusks), which are only present in males. Sex role description according to Kosztolányi et al.^17^ and Rosa et al.^19^.

In this work, we use *L. apterus* as a model organism for investigating sex-specific immune function in relation to reproductive investment. This iteroparous beetle is a good candidate for studies of sexual differences in resource allocation since it has a well-established sexual dimorphism and distinct, conventional sex roles (Fig. 1). They also have a brief and well-delineated active period, characterised by emerging from underground burrows in the spring to feed, find partners, and reproduce. They then return underground, where they spend most of the year in diapause^16,17^. We studied differential gene expression of immune-related genes to explore sex-specific intra-seasonal variation in immunity by sampling beetles of both sexes. We used a pseudo-longitudinal sampling framework where we sampled randomly selected adult individuals at consecutive sampling events. We conducted five consecutive sampling events (two weeks apart each; see Fig. 1) to capture the diversity in physiological and behavioural processes displayed by adult beetles as the period progresses. These five sampling events ultimately served as an indication of the relative sex roles undertaken by individuals throughout the active period. Because the active period in *L. apterus* could be energetically taxing and with varying levels of behavioural and physiological demands, we predicted that (1) the level of immune activation of adult beetles would fluctuate over time. Moreover, because females also allocate considerable resources into egg production, (2) we presumed that females would have an overall lower activation of immune-related genes than males^18^. Considering the two aforementioned predictions, namely, the sex role differences undertaken throughout the active period, (3) we predicted a significant interaction between sex and sampling date, where substantial sex differences in immune gene expression would be expected, particularly in the second half of the active period when sex role differences are most pronounced.

### Ethics statement

Sample collection from *L. apterus*, a species protected in Hungary, was approved by the Northern Hungarian Inspectorate for Environment Protection and Nature Conservation (No. 9007-8/2014).

## Results

### Transcriptome assembly and immune-related gene prediction

We found 10 genes (consisting of 19 transcripts) that were identified as AMPs, which together with the gene ontology (GO) annotation resulted in 296 immune-related genes (including 880 transcripts) that were expressed in *L. apterus* during the active period.

### Differential gene expression analysis

#### Overall effect of sex and sampling date on immune gene expression profiles

**Fig. 2:**
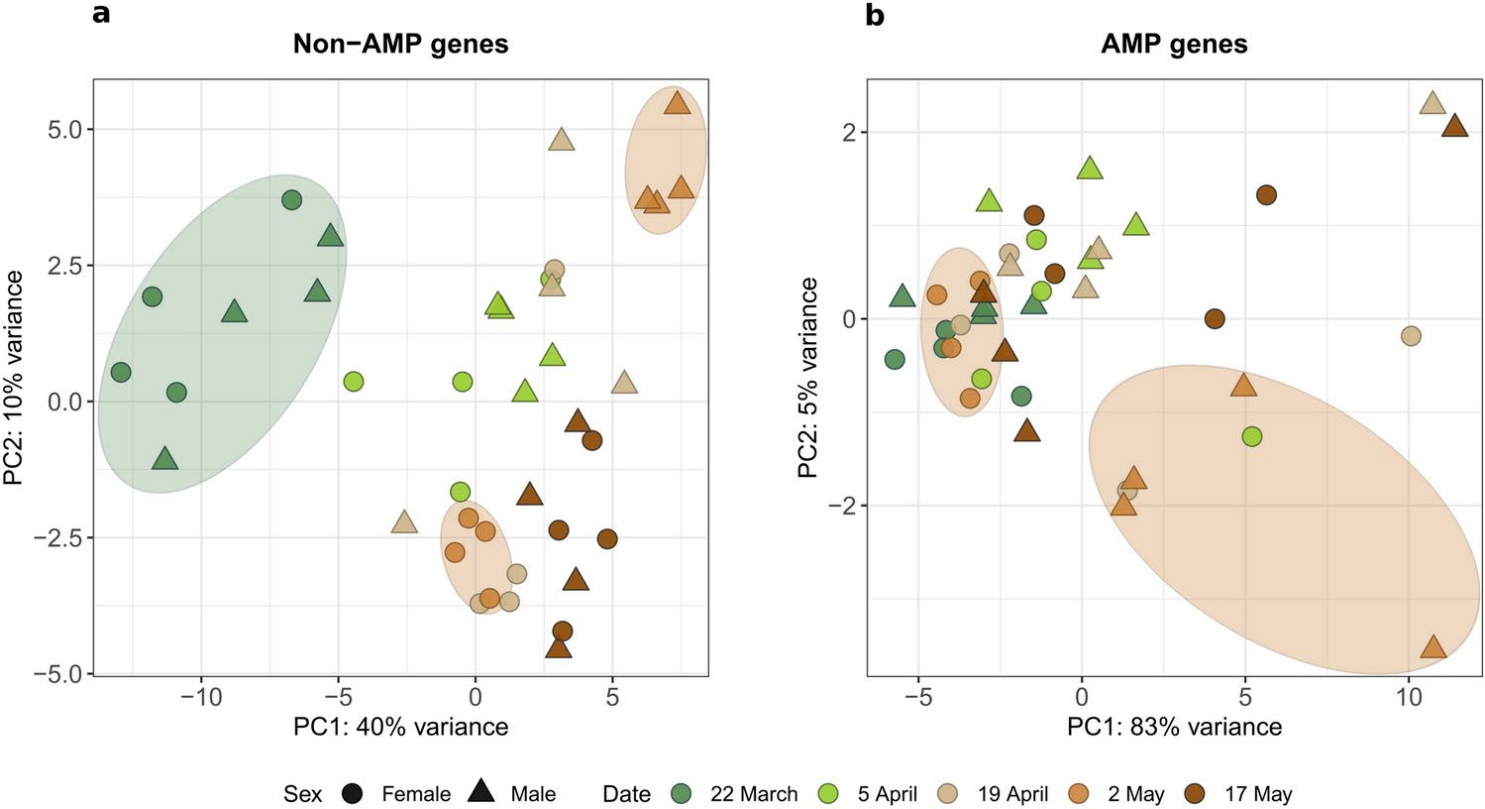
Principal component analysis (PCA) of male and female *Lethrus apterus* sampled on five consecutive dates within the active period. Plots were built based on expression values of (**a**) non-AMP immune genes and (**b**) AMP genes. Note the separation of the first date samples (22 March; highlighted in the confidence ellipse in green) from the other sampling dates for non-AMP genes, and the separate clustering of males and females on 2 May (confidence ellipse in orange) for both non-AMP and AMP genes.

Principal component analysis (PCA) revealed that for non-AMP genes there were two main groups, one consisting of only first-date samples and a second group including all other sampling dates (Fig. 2a). AMP genes showed no distinguishable groupings regarding sampling date (Fig. 2b). A notable clustering was detected in both non-AMP and AMP genes on 2 May (the fourth sampling date), where males and females clustered separately from each other, suggesting a high number of differentially expressed genes at this moment in the active period. This date corresponds to the middle of the parental care period based on female egg size^20^ (Fig. S1).

#### Sex-specific immune gene expression

**Fig. 3:**
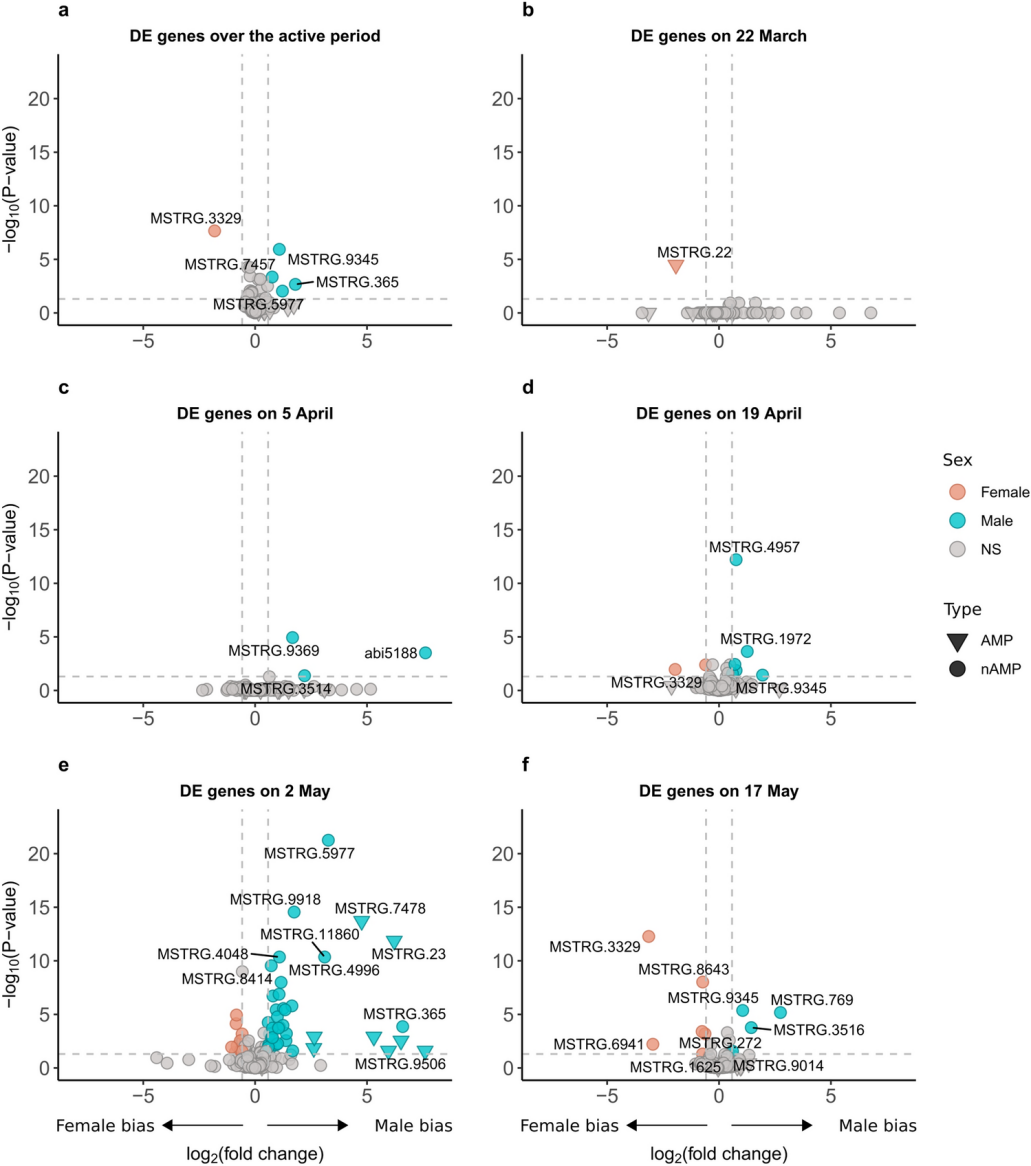
Volcano plots of the differentially expressed (DE) genes between the sexes. Overall sex difference in immune gene expression (**a**) and on each sampling date across the active period (**b**–**f**). Blue and orange coloured data points represent up-regulated genes where their positive and negative fold change represent a male and female bias, respectively. Grey points show non-significant (NS) genes, i.e. that failed to meet the cut-off threshold of significance (FDR < 0.05) and absolute log_2_ fold change value > 0.58 (i.e. fold change of 1.5 between the sexes). Upside-down triangles depict AMP genes whereas circles non-AMP immune-related genes.

We first investigated the overall immune gene expression differences between males and females throughout the active period. Then we addressed the sex differences by specific sampling date. When investigating all sampling occasions together, we found 24 immune genes having significant differences in their expression levels between the sexes. Four of these genes had at least 1.5-fold change in expression in males and only one in females when compared to the other (Fig. 3a). However, this difference in number of genes was not statistically significant (*P* > 0.05; Table 1). None of these genes were predicted as AMP (Table S3). Considering each sampling date separately, the number of differentially expressed genes between sexes varied among the sampling dates (Fig. 3b–f; χ^2^_4_ = 90.268, *P* < 0.001). The largest difference between the number of female- and male-biased genes occurred on 2 May (Fig. 3e; Table 1). Additionally, we note that only a few specific genes were consistently up-regulated on multiple but not necessarily consecutive sampling dates (see Table S3 for details).

**Table 1:** Number of differentially expressed immune genes in male and female *Lethrus apterus* throughout the active period and at each sampling date separately. Numbers in brackets represent genes that met both criteria of a false discovery rate (FDR) < 0.05 and had an absolute fold change value higher than 1.5. *P* values refer to Fisher’s exact test of the difference in gene up-regulation on the number of genes in brackets.

	Male-biased genes (up-regulated)	Female-biased genes (up-regulated)	*P* value
Overall	126 (4)	113 (1)	0.374
22 March	151 (0)	139 (1)	0.479
5 April	152 (3)	136 (0)	0.250
19 April	129 (6)	122 (2)	0.283
2 May	136 (38)	153 (10)	< 0.001
17 May	132 (4)	141 (6)	0.751

#### Seasonal changes in immune gene expression profile

Overall, there was a significant effect of sampling date on differential expression analysis across the active period of *L. apterus*. This analysis was conducted separately on females and males, revealing 142 and 137 genes with changing expression compared to the first sampling date, respectively. Based on their expression profiles, 13 and 11 clusters were produced, respectively (Fig. S2 and S3). According to their function, eight of these genes were predicted to be AMPs in females and only one in males. If only considering differentially expressed genes shared between females and males, there were 88 genes which were grouped into eight expression clusters. We note the often highly similar gene expression profiles in these shared genes (Fig. S4). One of these genes was predicted as AMP.

**Fig. 4:**
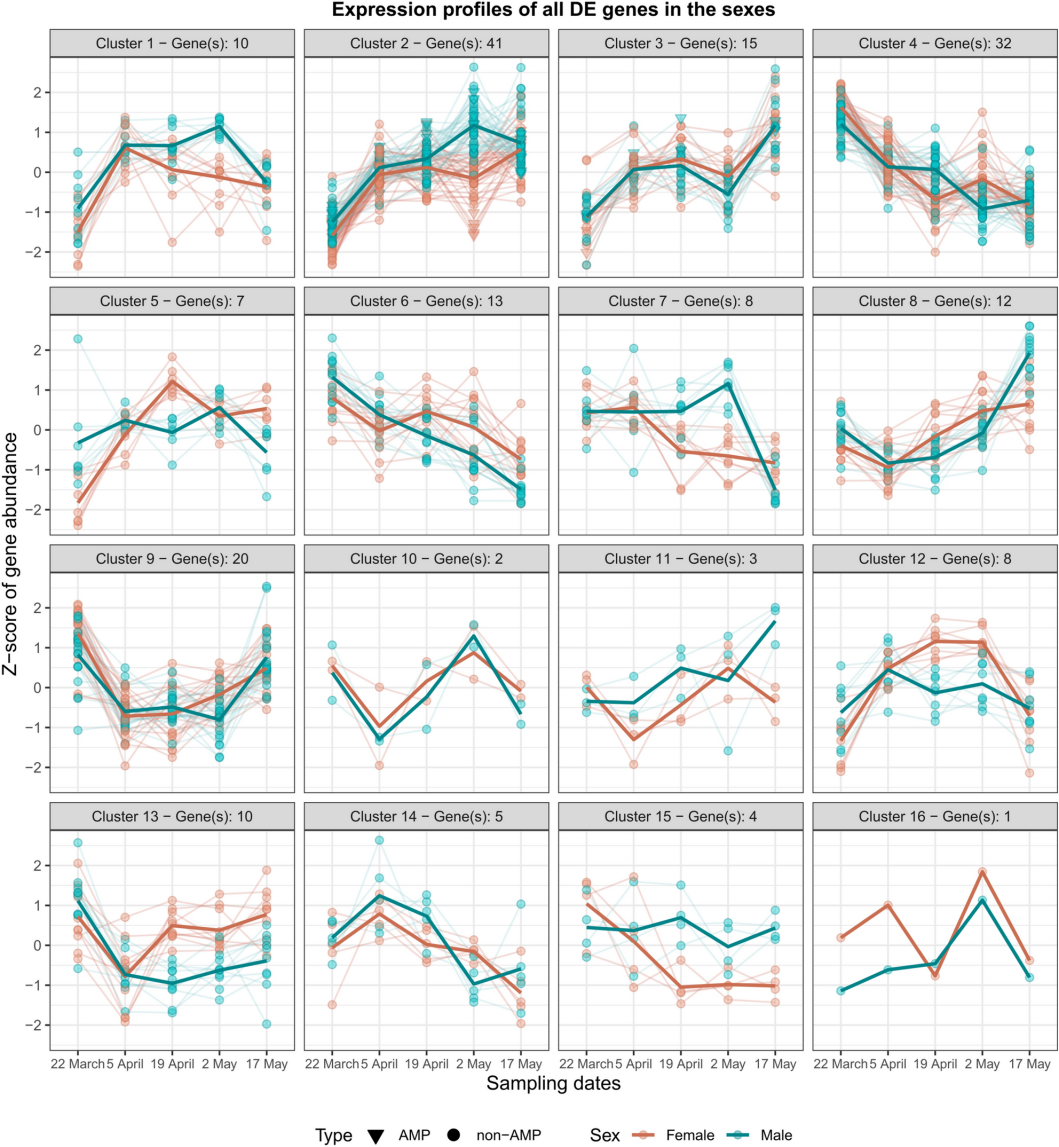
Expression profiles of all significantly differentially expressed immune genes across the active period of female and male *Lethrus apterus*. Gene abundance was estimated as Z-scores which are gene expression values centred and scaled by their mean and standard deviation, respectively. Positive values represent gene expression above average across samples, whereas negative values below average. Thick lines show the mean expression by sex in the given clusters. Number of genes in expression clusters are presented on top of each panel.

Finally, when all differentially expressed genes in at least one of the sexes across sampling dates were clustered according to their expression profile, 16 clusters were formed (Fig. 4). From these 191 genes, eight were AMP sequences (Table S3).

#### Sex-specific changes in immune gene expression over the active period

**Fig. 5:**
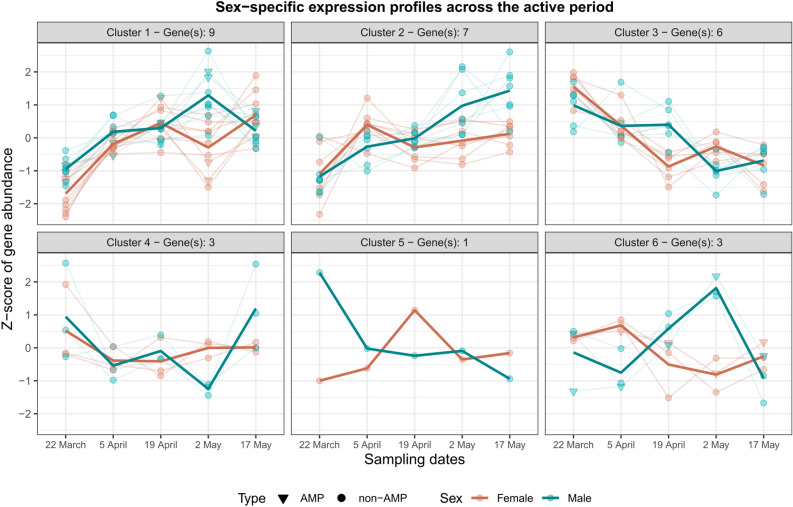
Sex-specific gene expression profiles across the active period in *Lethrus apterus*. Gene abundance was estimated as Z-scores which are gene expression values centred and scaled by their mean and standard deviation, respectively. Positive values represent gene expression above average across samples, whereas negative values below average. Number of genes consisting of the expression clusters are presented in the plot titles.

The model of the interaction between sex and sampling date resulted in 29 immune genes that showed a sex-specific change in expression over the active period, using the first sampling date as reference. Here, genes were arranged in six clusters based on their expression profile (Fig. 5). Most of the expression clusters had the highest sex differences either on 19 April or 2 May. Three genes were AMPs (Table S3).

## Discussion

The present study provides a thorough characterisation of the dynamics of sex-specific immune variation across the active period in *Lethrus apterus*. The use of sequential sampling —every two weeks from the beginning until the end of the active period— in combination with state-of-the-art transcriptomic methods allowed us showing that, as hypothesised, the immune gene expression presents male- and female-biased shifts throughout the active period.

Immune gene activation is a dynamic process that reflects on local pathogen pressures acting upon the host^21^. Although we had no information on potential parasite infection of individuals sampled, due to the lack of outliers in the PCAs in our results (Fig. 2), it might be assumed that the presented outcome represents baseline levels of immune activation in male and female beetles (i.e. homogenous pathogen burden among individuals at the moment of sampling).

We found differences in the level of immune gene expression throughout the five sampling dates, suggesting sequential increases and decreases in immune gene activation occurring within the active period in *L. apterus*. Drivers of immune variation could be numerous, but in insects previous work has described, for example, stress, food availability and social environment^22–25^. The aforementioned variables could also be contributing to some extent to our results, but we believe their influence to be marginal because our observations of important environmental factors, such as weather and food resources, remain rather stable throughout the active period in our study population. Instead, it is likely that the differences found between sampling dates are largely attributed to the duties undertaken during the active period, i.e. sex roles. This is because, soon after emergence, the beetles engage in increasingly demanding activities that could result in immunosuppression. Immunocompetence may be temporarily suppressed during certain life stages to allocate resources for reproductive investment^4,6,26^. Studies addressing intra-seasonal changes in immunity are rather scant in insects, but our results are in line with part of the findings presented in a previous study on *L. apterus*, showing presence of seasonality in some aspects of immune defence^12^. In that study, Kiss et al.^12^ measured and compared the immune responses of the sexes at early and late time points in the season. Although their work focused on higher-level immune responses rather than gene expression, females exhibited a higher haemolymphal capacity to inhibit bacterial growth compared to that of males. Another study on burying beetles (*Nicrophorus orbicollis*) also demonstrated changes in immune activity within the breeding period, revealing an up-regulation in encapsulation response and lytic activity in anal exudates upon finding a carcass, that remained elevated throughout the entire parental care period^27^. This improved immunity amid the breeding period is intriguing. However, we note that the life style of *N. orbicollis* is prominently different from our study model, because the former is attracted to carrion for reproduction and feeding, suggesting from that point onwards, an elevated exposure to pathogens^28^. Moreover, Steiger et al.^27^ argues that the surprising lack of trade-offs between immunity and reproduction could be attributed to an unfettered access to protein-rich food.

Sex-specific gene expression analysis showed that, overall, across the active period there was a male bias in the up-regulation of the immune-related genes but this difference was not statistically significant. One possibility is that, despite males and females fulfilling considerably different roles at specific moments within the active period, overall, the total investment incurred by each sex is rather comparable. This is also supported by our findings on temporal gene expression profiles that were rather similar between the sexes when looking at the entire active period (Fig. S4). Our findings differ from a previous meta-analysis in insects by Nunn et al.^29^ that showed a female-biased immunocompetence, but coincides with the findings of another recent meta-analysis, finding an overall lack of difference between the sexes^30^. However, Kelly et al.^30^ did find female-biases in specific immune parameters. In this context, it is possible that the partial agreement of our findings is due to differences in immune status when investigating different periods within the yearly cycle. Sampling design could also play a role, that is, cross-sectional versus pseudo-longitudinal sampling. Additionally, studies usually address immunity via a selection of different immune markers, whose outcomes should be interpreted individually because their function tends to be highly specific and not comparable with other markers^12,31,32^.

We found no genes showing consistent differential expression across the five sampling dates between sexes. Instead, a few immune genes appeared to be recurrently up- or down-regulated on specific, but not consecutive, dates. This could result from external factors such as agonistic behaviour toward conspecifics or other insects (possibly due to injuries) or feeding from specific food sources that could influence immune gene activation^33^. We highlight genes including the growth/differentiation factor 8 that had higher expression in females at the beginning, middle and end of the season, and a gene predicted as filamin A, which had a higher expression in males on most sampling dates. Since these are single genes and not molecular pathways, it is difficult to propose why they are upregulated at specific moments. Although growth/differentiation factor 8 had not yet been associated with insect immunity, our GO annotation method predicted it to be a positive regulator of immune system processes. Additionally, filamin A was showed to have antimicrobial activity but only in echinoderms to date^34^.

When we analysed the interaction between sex and sampling date, we found prominent sex differences in immune gene expression on specific dates. As mentioned before, many factors are recognised as immune modulators in insects, however, sex-specific immune variation is generally mediated by alternative variables, including mating status and reproductive investment^12,18,35^. On date four (2 May), we saw the largest number of differentially expressed immune genes, including a strong down-regulation in AMP coding genes in females (see Fig. 4—Cluster 2; Fig. S2—Clusters 3 and 4). These changes coincidentally matched the peak of the active period in *L. apterus* (Fig. 1 and S1; also shown in Nagy et al.^20^). On this date, sex roles differ substantially from the other dates, with females diligently collecting leaves to provide for their previously laid eggs. Males, on the other hand, typically stay at the entrance of the burrow to defend against potential rivals or predators. This differential behavioural investment between the sexes could play a role in mediating the observed sex differences in immune gene expression. As mentioned earlier, several studies have shown trade-offs between resource allocation in reproduction and immune responses^36^, but examples of these trade-offs occurring in a sex-specific manner are scarce. In insects, there is substantial evidence showing that females undergo immunosuppression, particularly during events that take place during reproduction such as egg production (reviewed by Schwenke et al.^37^). Because males are typically less involved in reproduction, it is assumed that their immune function is not compromised to the extent of females. Another explanation is that males get injured more often as a consequence of frequent fighting^38^, so they need a higher level of baseline immunity to fight pathogens. On the other hand, juvenile hormone is known to have an immunosuppressive effect in both sexes in insects, and it could be mediating part of our results^10,39^. Unfortunately, there is no information on this hormonal mediator in *L. apterus* which hinders drawing further conclusion. Interestingly, the third sampling date (19 April), that is, two weeks prior the peak of the breeding season, also showed notable relevance (Fig. 5—Clusters 3 and 5). Based on the GO annotation, these genes were mainly involved in the peptidoglycan recognition and encapsulation process, based on studies on other taxa such as fruit flies and silkworms^40,41^.

Among the variety of mechanisms animals exploit to improve their survival, the immune system is characterised as highly effective, complex and its competence can be influenced by numerous variables such as seasonality. The study of *L. apterus* offers an advantage over other model systems, as this beetle spends most of the year inactive in diapause underground, which may reduce interference by potential confounding factors. However, among the caveats of studying changes in gene expression in a natural population, we highlight fluctuating environmental factors such as temperature and precipitation^42^. We should also consider biotic factors including pathogen infection or physical injury^43,44^. Future works should put emphasis on controlling such potentially confounding variables in the *L. apterus* model system. Our results show that the magnitude of immune activation fluctuates across their active period, with important differences between the immune gene expression of the sexes. While we showed that sex-specific immunity varies with sampling date, it is still unknown to what extent each specific behaviour conducted by the beetles influences immune activity. Our assumptions regarding immunological trade-offs were based on literature^10,37^, however, we note that future experimental studies can shed light on the immunological cost of reproduction of males and females in *L. apterus*. Lastly, our work highlights the importance of sequential sampling schemes over cross-sectional sampling to gain a more comprehensive understanding of ecological dynamics in the wild.

## Materials and methods

### The model organism

*Lethrus apterus* is a sexually dimorphic beetle with distinct sex roles (Fig. 1). Males possess two ventral mandibular processes (tusks) used for intra-sexual competition (Fig. 1). Individuals hibernate underground for most of the year and are only active once they have emerged to the surface, between March and June. At the beginning of the active period, in early spring, individuals dig a 10–20 cm-deep tunnel which serves as a shelter before they find a partner. Paired beetles prepare a 50–90 cm-deep burrow, terminating in six to eight brood chambers, each of them serving as nest for a single developing egg^7,17,45,46^. Eggs are relatively large, laid sequentially and provisioned with green leaves the parents gather in the area surrounding the burrow (i.e. an area of about 3.5 m^2^, Frantsevich et al.^16^). Once the egg chamber has enough leaves, they close it and repeat this process with the next chamber. Food provisioning is arduous and predominantly done by the female, while males only occasionally collect leaves, instead spending most of their time guarding the burrow from intruders^17,19,47^.

### Sample collection

Sample collection was carried out in spring of 2019, during the active period of *L. apterus* in a dense population (approx. 6000–7000 individuals, J.L-K. pers. obs.) near the village of Susa, Hungary (48°16′27″N, 20°15′08″E). Individuals were captured in an area of approx. 60 × 20 m. We collected four males and four females on each date, corresponding to five dates and covering the whole active period (n = 40): 22 March, 5 and 19 April, 2 and 17 May (Fig. 1). From each individual collected, we only extracted head tissue (all content from inside the head capsule i.e., muscle, cells of the nervous system, haemolymph and fat tissue) in order to reduce any possible contamination from intestinal content because beetles are continuously feeding during the active period. At the moment of collection, we also recorded the sex and the actual observed activity of the individuals (three possible categories: leaf collection, out of the nest, in the nest). Head tissue were immediately put in 600 μL RNA/DNA Shield buffer (Zymo Research, USA) and stored at −20 °C until RNA extraction. The abdomen was stored in 70% ethanol and the genitals were dissected. In particular, gonad size was determined in order to confirm breeding status at the population level^20,48,49^. Developing and mature egg size increased in the second half of the season and peaked on 2 May (linear regression model, *P* < 0.001; Fig. S1). This is in line with previous observations on changing leaf collecting activity of females upon the beginning of the parental care period^20^, which demonstrates that our sampling window is a good representation of the reproductive changes. Testis size did not significantly vary as season progressed (*P* > 0.05; Fig S1; but see^12,50^). We only collected tissue samples from beetles in good apparent physical condition (e.g., no missing appendages, no chitin decolouration, etc.), though parasite infection was not recorded.

### RNA isolation and Illumina sequencing

Total RNA was isolated from the samples using TRI Reagent Solution (Thermo Fisher Scientific, USA) following the manufacturer’s directions. RNA concentration was measured using NanoDrop 1000 Spectrophotometer (Thermo Fisher Scientific, USA) and RNA integrity was assessed through 1% agarose gel electrophoresis (see further details in supplementary methods). cDNA library preparation, purification and 150 bp paired-end (PE) sequencing on Illumina HiSeq 4000 platform were conducted by Novogene Co. (Beijing, China).

### Transcriptome assembly and functional annotation

Quality of the raw reads was checked using FastQC v0.11.8^51^. Adapter sequences and low quality bases were removed using Trimmomatic v0.39^52^. Cleaned reads were mapped to an improved version of the *L. apterus* genome sequence assembled using next generation and third generation sequencing data (submitted to GenBank) with HISAT2 v2.2.1^53^. Based on the read alignments, we performed reference guided transcriptome assembly using StringTie software v2.1.4^54^ and following the recommendations by Pertea et al.^55^. More details are provided in supplementary methods.

Sequences of the assembled transcripts were obtained from the reference genome based on the general transfer format (GTF) file of the merged set of transcripts (see above) using gffreads from general feature format (GFF) utilities v0.12.3^56^. Potential open reading frames (ORFs) were predicted using TransDecoder v5.7.1^57^, and the predicted protein sequences were functionally annotated using the Pannzer2 webserver^58^. Results were restricted to fit gene ontology (GO^59)^ classes having non-IEA (i.e. manually reviewed) annotations in Arthropoda. Sequences were accepted as coding for immune proteins if they were classified as immune-related GO with at least one record on the FlyBase^60^, suggesting its presence in arthropods (see full list in Table S1).

### Antimicrobial peptide (AMP) prediction

We searched for AMP coding genes among the potential ORFs using the HMMER software package v3.3.2^61^ which uses hidden Markov models (HMM) to find homologs based on a user-defined sequence profile database. All AMP groups of beetles^62^ (Table S2), namely defensins, tenecins, holotricins, coprisins, sarcotoxins, cecropins and attacins were used for finding potential AMPs in *L. apterus* (see further details in supplementary methods). Sequences of the different AMP groups were used separately for building HMM profiles. As suggested by the HMMER package, amino acid sequences of an AMP group were aligned using MUSCLE v3.8.1551^63^ with its default parameters. These alignments were converted into Stockholm format. The command ‘hmmsearch’ with an E-value of 0.001 was applied to find homologous sequences among the predicted ORFs of *L. apterus*. Potential AMPs were validated using the hidden Markov model ADAM tool (accessed on 17 October 2023; Lee et al.^64^) which was found to be the best method for AMP prediction in coleopteran species^65^.

### Analysis of differentially expressed genes

Gene counts for the differential gene expression (DGE) analyses were obtained using the prepDE.py script provided by StringTie v2.1.4^54^. To examine the overall expression variation of the samples according to sex, date and activity, we conducted principal component analysis (PCA) using the gene expression matrix as input. Importantly, PCA showed no outliers suggesting homogenous sampling in the field. DGE analyses of immune and AMP genes were performed using the DESeq function of the DESeq2 Bioconductor package v1.34.0^66^. The DESeq function fits a negative binomial generalised linear model (GLM) to the count data in order to model the relationship between the expression of each gene and experimental conditions. Significance was tested using likelihood ratio tests from the DESeq function, which compared full versus reduced models. Three separate sets of models were built in order to investigate the effect of sex and the effect of sampling date. All models were corrected for activity (of the beetle at the moment of capture; see above), which was considered as a batch effect^66^, and had a false discovery rate (FDR) of alpha < 0.05. First, the effect of (1) sex was studied by building full models that included sex, sampling date and activity, which was compared to a reduced model that excluded sex. Pairwise comparisons between the sexes were also performed for each sampling date separately where the full models with sex and activity were compared to the reduced model without sex. In these tests, we added an additional cut-off value of 1.5 fold change for the differential gene expression between the sexes, which is a sensible threshold for natural populations to capture smaller changes besides the big differences in expression levels^67^. Change in the number of genes having statistically significant expression differences between sexes at the five sampling occasions was tested with chi-square tests to examine the distribution of sex differences throughout the active period. Further, we used Fisher’s exact tests for the overall sample and for each sampling date separately to find the sampling dates in which the number of female- and male-biased genes differed significantly. Second, to investigate the effect of (2) sampling date (i.e., seasonality), we built a separate model for females and one for males. Here, full models had activity and sampling date and reduced models excluded sampling date. Lastly, (3) the two-way interaction between sex and sampling date was studied by building models with sex, date, their interaction and activity, and the reduced model was missing the interaction term.

Differentially expressed immune genes that showed statistically significant differences in the prior analysis were visualised using the degPatterns function from the DEGReport Bioconductor package v1.30.0^68^. This function clusters the genes according to their similarities in expression pattern (i.e. expression profiles) over the active period. It operates by first merging samples belonging to the same time point (date) and group (sex). The mean expression values of the genes in a merged group are used to calculate the pairwise correlations between all the genes, from which a distance matrix is created. Clustering is performed based on this matrix using the divisive hierarchical clustering (DIANA algorithm).

## Supplementary Information

Supplementary Information 1.

Supplementary Information 2.

## Data Availability

Illumina sequencing reads used to assemble the transcriptome have been submitted to the Sequence Read Archive (SRA) of the National Centre for Biotechnology Information (NCBI) under the accession code PRJNA1169319. Genome assembly and annotation used for the genome-guided transcriptome assembly are available on Zenodo.org (10.5281/zenodo.13905567).
