# Supplementary material for: Shifts in sex-specific immune gene expression in a beetle with parental care
Source: Sci Rep. 2025 Mar 29;15:10930. doi: 10.1038/s41598-025-95268-4 (PMC11954957; doi:10.1038/s41598-025-95268-4)
Supplement: Supplementary file 1 — Supplementary Information 1. [file 41598_2025_95268_MOESM1_ESM.docx]

**Supplementary material:**

**Shifts in sex-specific immune gene expression in a beetle with parental care**


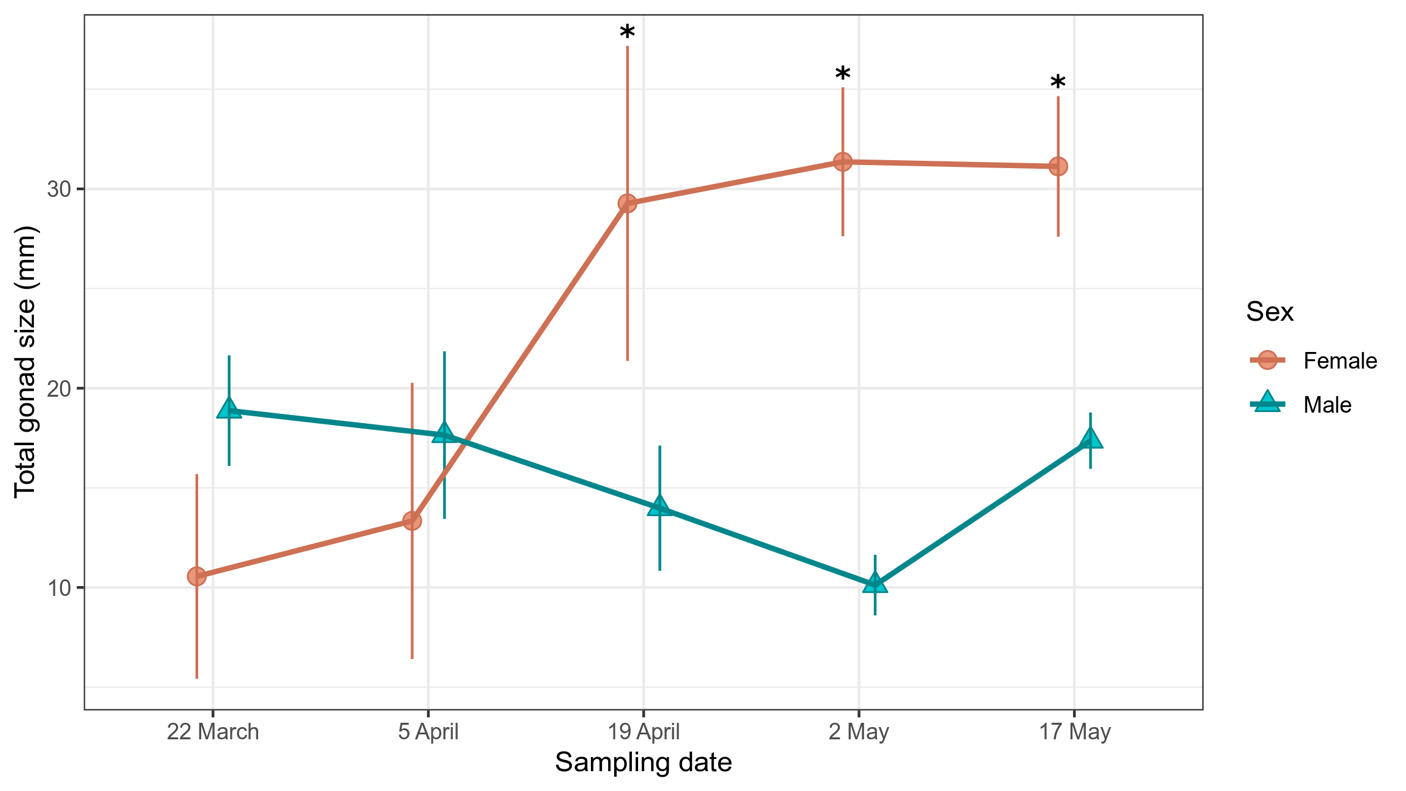


**Figure S1.** **Variation (mean ± standard deviation) in egg and testes (follicles) size in females and males of *Lethrus apterus* over the active period.** Significant differences compared to the first sampling date are marked with asterisks. Note that the fourth sampling date represented the points of lowest and highest size for males and females, respectively.


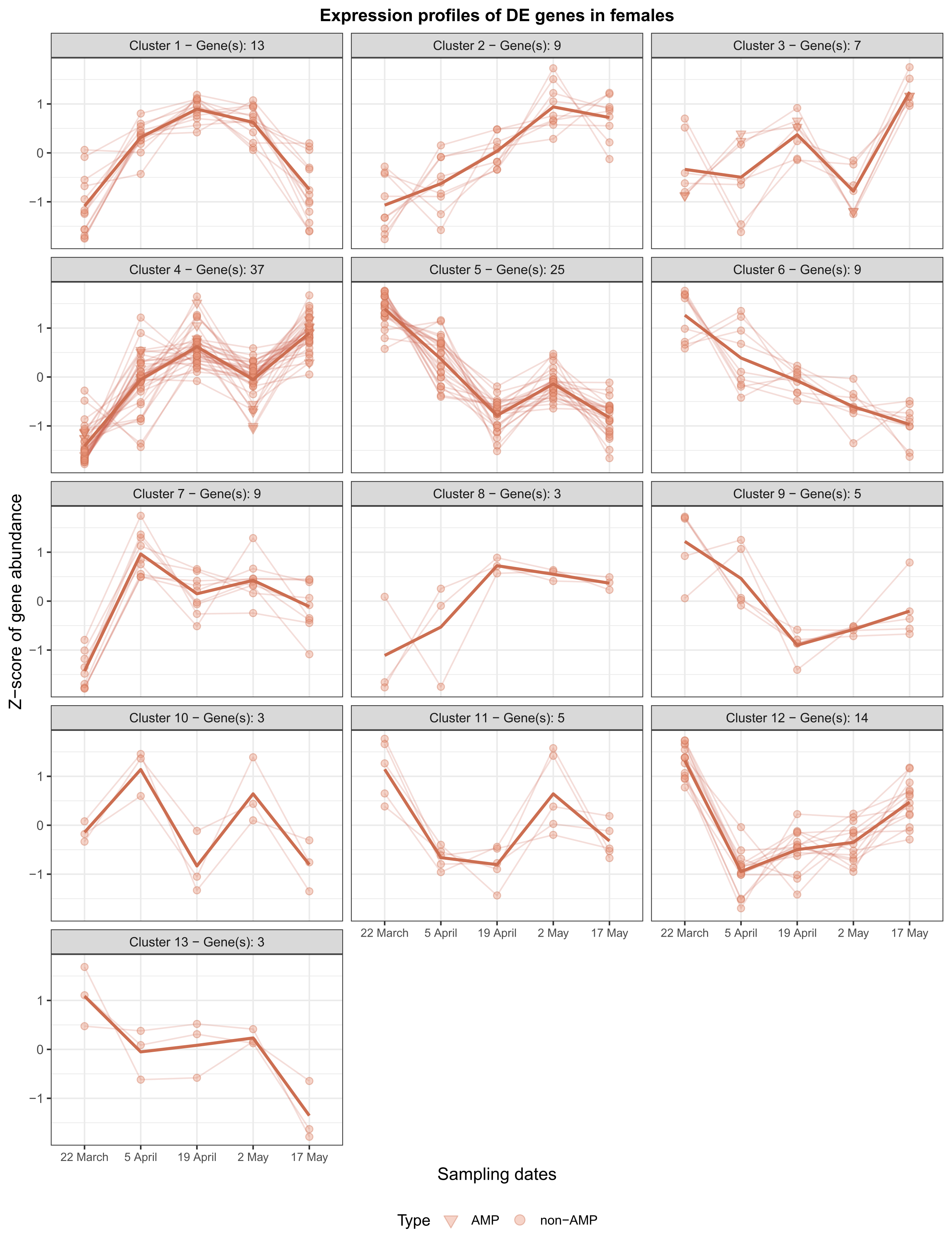
**Figure S2.** **Expression profile of genes changing significantly in female *Lethrus apterus* individuals over the active period.** Gene abundance was estimated as Z-scores which are gene expression values centred and scaled by their mean and standard deviation, respectively. Positive values represent gene expression above average across samples, whereas negative values below average. Number of genes consisting the expression clusters are presented in the panel titles.


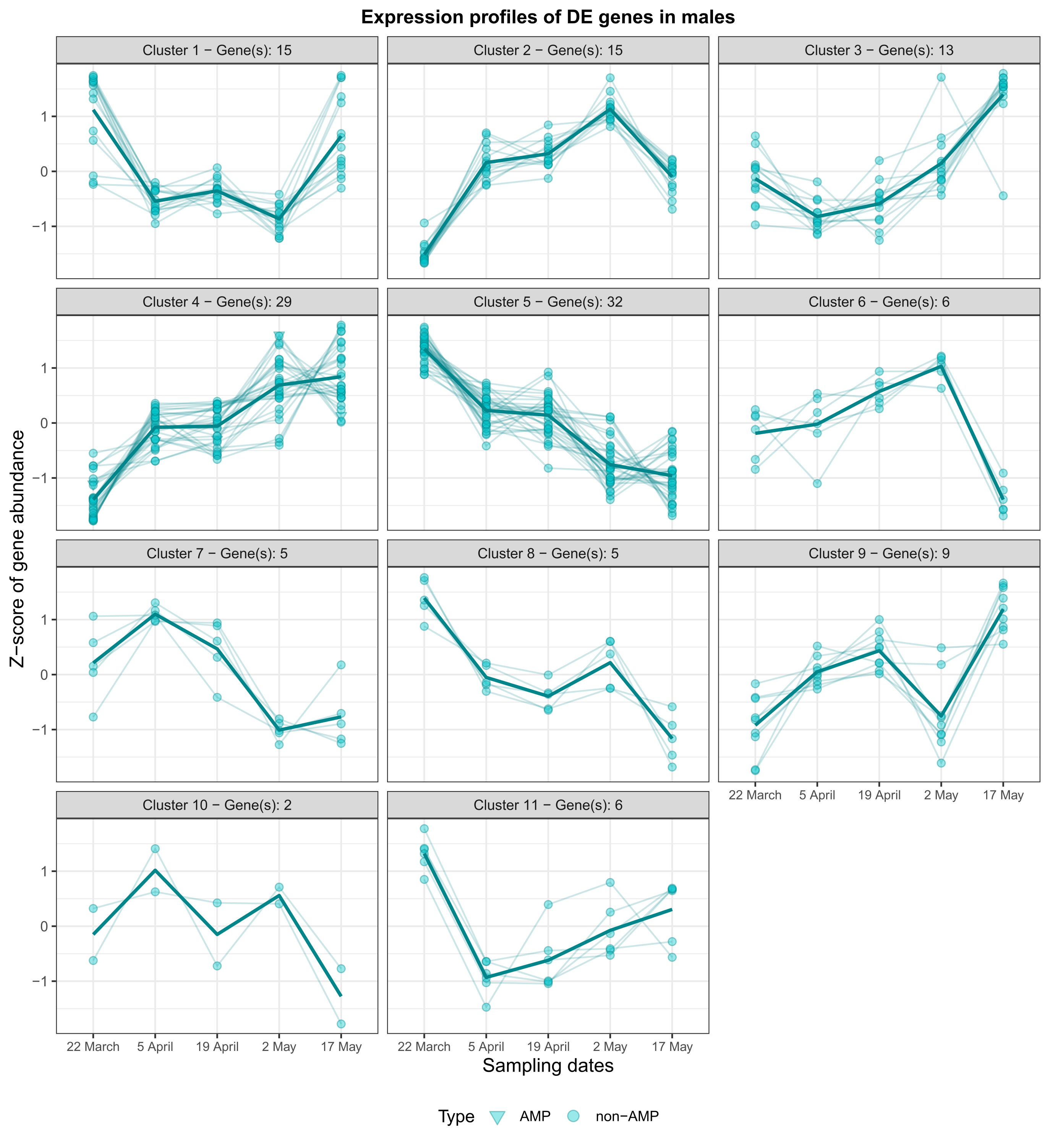
**Figure S3.** **Expression profile of significantly changing genes in male *Lethrus apterus* individuals during the active period.** Gene abundance was estimated as Z-scores which are gene expression values centred and scaled by their mean and standard deviation, respectively. Positive values represent gene expression above average across samples, whereas negative values below average. Number of genes consisting the expression clusters are presented in the plot titles.


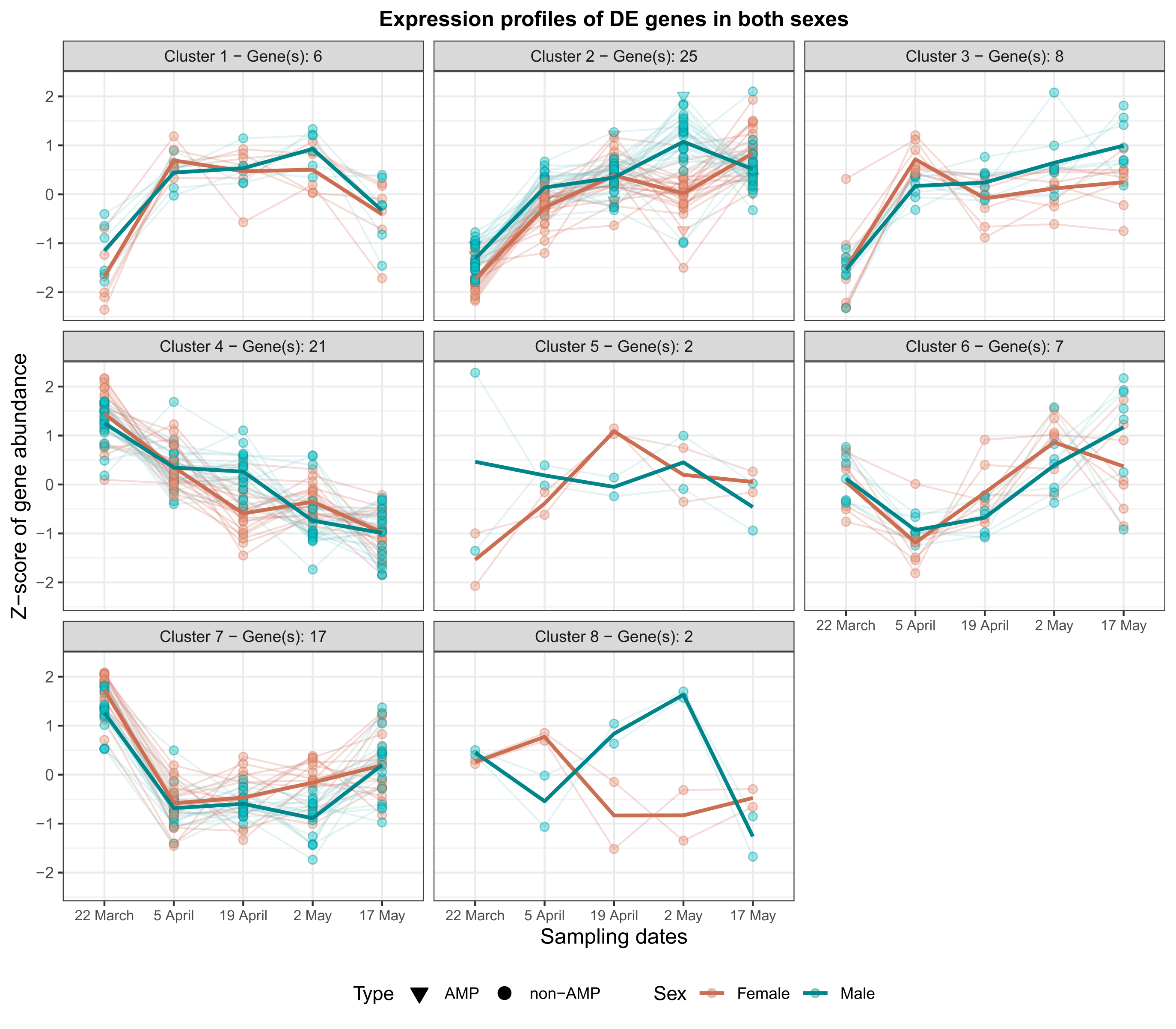
**Figure S4.** **Expression profile of genes that were significantly changing in both females and males over the active period of *Lethrus apterus*.** Orange lines and points show female, whereas blue lines and rectangles refer to male expression changes. Gene abundance was estimated as Z-scores which are gene expression values centred and scaled by their mean and standard deviation, respectively. Positive values represent gene expression above average across samples, whereas negative values below average. Number of genes consisting the expression clusters are presented in the plot titles.

**SUPPLEMENTARY METHODS**

**RNA isolation and Illumina sequencing**

Total RNA was isolated from the samples using TRI Reagent Solution (Thermo Fisher Scientific, USA) following the manufacturer’s directions. Pellets were diluted in 30 μL RNase-free water. Concentration of the samples was measured using NanoDrop 1000 Spectrophotometer (Thermo Fisher Scientific, USA) and RNA integrity was assessed through 1% agarose gel electrophoresis. RNA samples were stored at -20°C until sequencing. cDNA library preparation, purification and 150 bp paired-end (PE) sequencing on Illumina HiSeq 4000 platform were conducted by Novogene Co. (Beijing, China).

**Transcriptome assembly and functional annotation**

Quality of the raw reads was checked using FastQC v0.11.8^[1]^. Adapter sequences and low quality bases were removed using Trimmomatic v0.39^[2]^ with TruSeq3-PE-2.fa adapters, and the following parameters: “ILLUMINACLIP:2:30:10 LEADING:5 TRAILING:5 SLIDINGWINDOW:4:25 MINLEN:40”. Cleaned reads were mapped to an improved version of the *L. apterus* genome sequence assembled using next generation and third generation sequencing data (submitted to GenBank) with HISAT2 v2.2.1^[3]^. Based on the read alignments, we performed reference guided transcriptome assembly using StringTie software v2.1.4^[4]^. As recommended by Pertea et al.^[5]^, we first assembled the transcriptomes of each sample then merged the transcripts of all samples into a uniform, non-redundant set of transcripts (--merge flag) which was used as reference for re-estimation of transcript abundances (with -e option) in each sample.

Sequences of the assembled transcripts were obtained from the reference genome based on the general transfer format (GTF) file of the merged set of transcripts (see above) using gffreads from general feature format (GFF) utilities v0.12.3^[6]^. Potential open reading frames (ORFs) were predicted using TransDecoder v5.7.1^[7]^, and the predicted protein sequences were functionally annotated using the Pannzer2 webserver^[8]^. Results were restricted to fit gene ontology (GO^[9]^) classes having non-IEA (i.e. manually reviewed) annotations in Arthropoda. Sequences were accepted as coding for immune proteins if they were classified as immune-related GO with at least one record on the FlyBase^[10]^, suggesting its presence in arthropods (see full list in Table S1).

**Antimicrobial peptide (AMP) prediction**

We searched for AMP coding genes among the potential ORFs using the HMMER software package v3.3.2^[11]^ which uses hidden Markov models (HMM) to find homologs based on a user-defined sequence profile database. All AMP groups of beetles^[12]^ (Table S2), namely defensins, tenecins, holotricins, coprisins, sarcotoxins, cecropins and attacins were used for finding potential AMPs in *L. apterus*. The following search terms were used to find AMPs of coleopteran species in the UniProt database (downloaded on 22^th^ August 2023): “(((protein_name:defensin) OR (protein_name:tenecin) OR (protein_name:holotricin) OR (protein_name:coprisin) OR (protein_name:cecropin) OR (protein_name:sarcotoxin) OR (protein_name:attacin)) OR (protein_name:coleoptericin)) AND (taxonomy_id:7041)) NOT putative”. Sequences of the different AMP groups were used separately for building HMM profiles. As suggested by the HMMER package, amino acid sequences of an AMP group were aligned using MUSCLE v3.8.1551^[13]^ with its default parameters. These alignments were converted into Stockholm format with the ‘esl-reformat’ command provided by the HMMER package, which were then used by the ‘hmmbuild’ command to create peptide profiles. The command ‘hmmsearch’ with an E-value of 0.001 was applied to find homologous sequences among the predicted ORFs of *L. apterus*. Potential AMPs were validated using the hidden Markov model ADAM tool (accessed on 17^th^ October 2023; Lee et al.^[14]^) which was found to be the best method for AMP prediction in coleopteran species^[15]^.

**References**

1. Andrews, S. FastQC: A Quality Control Tool for High Throughput Sequence Data. [Available online at: http://www.bioinformatics.babraham.ac.uk/projects/fastqc/]. (2010).

2. Bolger, A. M., Lohse, M. & Usadel, B. Trimmomatic: a flexible trimmer for Illumina sequence data. *Bioinformatics* **30**, 2114–2120 (2014).

3. Kim, D., Paggi, J. M., Park, C., Bennett, C. & Salzberg, S. L. Graph-based genome alignment and genotyping with HISAT2 and HISAT-genotype. *Nat Biotechnol* **37**, 907–915 (2019).

4. Pertea, M. *et al.* StringTie enables improved reconstruction of a transcriptome from RNA-seq reads. *Nat Biotechnol* **33**, 290–295 (2015).

5. Pertea, M., Kim, D., Pertea, G. M., Leek, J. T. & Salzberg, S. L. Transcript-level expression analysis of RNA-seq experiments with HISAT, StringTie and Ballgown. *Nat Protoc* **11**, 1650–1667 (2016).

6. Pertea, G. & Pertea, M. GFF Utilities: GffRead and GffCompare. *F1000Res* **9**, ISCB Comm J-304 (2020).

7. Haas, B. J. TransDecoder. [Available online at: https://github.com/TransDecoder/TransDecoder]. (2023).

8. Törönen, P. & Holm, L. PANNZER-A practical tool for protein function prediction. *Protein Sci* **31**, 118–128 (2022).

9. Ashburner, M. *et al.* Gene ontology: tool for the unification of biology. The Gene Ontology Consortium. *Nat Genet* **25**, 25–29 (2000).

10. Gramates, L. S. *et al.* FlyBase: a guided tour of highlighted features. *Genetics* **220**, iyac035 (2022).

11. Eddy, S. R. Accelerated Profile HMM Searches. *PLoS Comput Biol* **7**, e1002195 (2011).

12. Yi, H.-Y., Chowdhury, M., Huang, Y.-D. & Yu, X.-Q. Insect Antimicrobial Peptides and Their Applications. *Appl Microbiol Biotechnol* **98**, 5807–5822 (2014).

13. Edgar, R. C. MUSCLE: multiple sequence alignment with high accuracy and high throughput. *Nucleic Acids Res* **32**, 1792–1797 (2004).

14. Lee, H.-T., Lee, C.-C., Yang, J.-R., Lai, J. Z. C. & Chang, K. Y. A Large-Scale Structural Classification of Antimicrobial Peptides. *Biomed Res Int* **2015**, 475062 (2015).

15. Rádai, Z., Kiss, J. & Nagy, N. A. Taxonomic bias in AMP prediction of invertebrate peptides. *Sci Rep* **11**, 17924 (2021).
